# A novel 3D-printed hybrid simulation model for robotic-assisted kidney transplantation (RAKT)

**DOI:** 10.1007/s11701-018-0780-y

**Published:** 2018-01-27

**Authors:** Raphael Uwechue, Petrut Gogalniceanu, Nicos Kessaris, Nick Byrne, Pankaj Chandak, Jonathon Olsburgh, Kamran Ahmed, Nizam Mamode, Ioannis Loukopoulos

**Affiliations:** 1 Department of Transplantation, Guy’s Hospital, Renal Offices, 6th Floor Borough Wing, Great Maze Pond, London, SE1 9RT UK; 2 Department of Medical Physics, Guy’s and St Thomas’ NHS Foundation Trust, London, UK; 3 School of Biomedical Engineering and Imaging Sciences, King’s College London, London, UK; 4 MRC Centre for Transplantation, NIHR Biomedical Research Centre, King’s Health Partners, King’s College London, London, UK; 5 Department of Urology, Guy’s and St Thomas’ NHS Foundation Trust, London, UK

**Keywords:** Kidney transplantation, Simulation, Training, Robotic

## Abstract

Robotic-assisted kidney transplantation (RAKT) offers key benefits for patients that have been demonstrated in several studies. A barrier to the wider uptake of RAKT is surgical skill acquisition. This is exacerbated by the challenges of modern surgery with reduced surgical training time, patient safety concerns and financial pressures. Simulation is a well-established method of developing surgical skill in a safe and controlled environment away from the patient. We have developed a 3D printed simulation model for the key step of the kidney transplant operation which is the vascular anastomosis. The model is anatomically accurate, based on the CT scans of patients and it incorporates deceased donor vascular tissue. Crucially, it was developed to be used in the robotic operating theatre with the operating robot to enhance its fidelity. It is portable and relatively inexpensive when compared with other forms of simulation such as virtual reality or animal lab training. It thus has the potential of being more accessible as a training tool for the safe acquisition of RAKT specific skills. We demonstrate this model here.

Robotic-assisted kidney transplantation (RAKT) may provide enhanced patient recovery with comparable outcomes to standard open surgery. In addition, RAKT can increase access to kidney transplantation for specific end stage renal disease (ESRD) patients with morbid obesity [1]. The robotic platform provides high resolution three dimensional (3D) operative views enhancing stereotactic vision and improved depth perception [2], as well as superior instrument handling with a vastly increased range of movement [3] compared to laparoscopic surgery. Consequently, RAKT is progressively expanding and showing promising results in centers of excellence [4, 5].

In robotic surgery, as in all other surgical approaches, patient safety is paramount. Skill acquisition for surgery in general, but particularly in robotic surgery, is hampered by time pressure [6], service provision and financial imperatives [7]. Simulation is a well-established method of teaching operative skills, reducing learning curves and providing transferable skills to the operating theatre [8] that improve operator performance [9]. Current simulation training is based on virtual reality simulators, in vivo animal models and cadaveric models [10]. These models remain costly and difficult to access. In addition, teams need to practice non-technical skills including the robot setup, patient positioning and interacting dynamically with the robotic system to allow surgeons to operate successfully. These skills can be improved through simulation training [11].

3D-printing or rapid prototyping is a form of additive manufacturing technology that now has several recognized applications in the medical field including the creation of anatomical models for surgical planning, patient-specific surgical guides and custom prosthetic implants, as well as in medical education [12]. 3D printed models have now been demonstrated to show a high degree of anatomical accuracy, with implicit application as simulation training tools for the acquisition of technical skills [13] (Fig. 1).

**Fig. 1:**
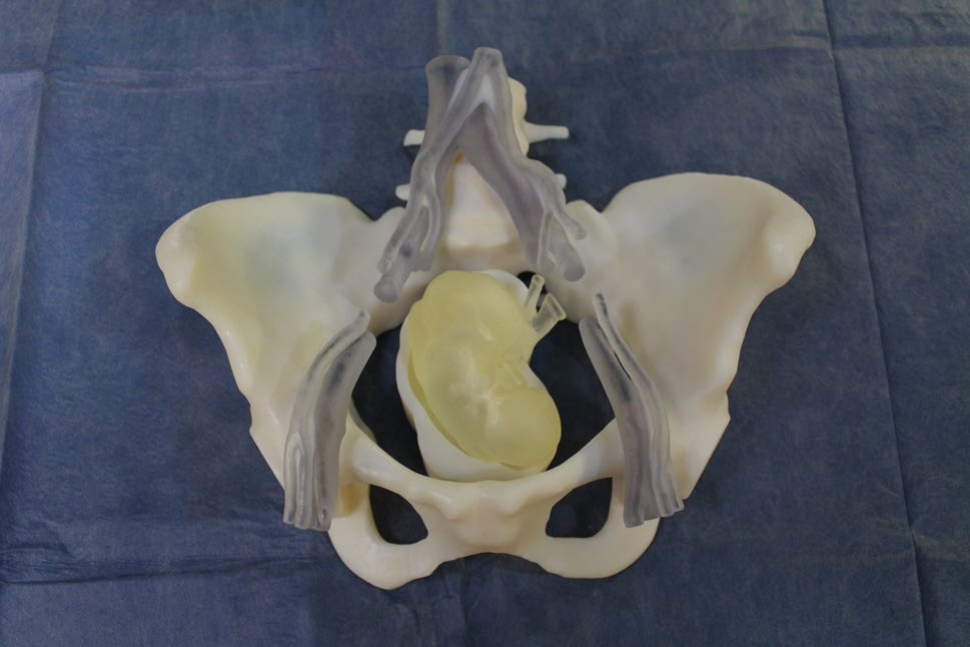
Prosthetic cradles

A high-volume tertiary abdominal transplant unit in the UK planned to introduce a RAKT programme. To facilitate the new procedure, we created a hybrid simulation training model combining a 3D printed-moulage, integrated with deceased donor vessels. The purpose of the model was to optimise the surgeons training in vascular anastomoses in a timed and reproducible manner to allow objective assessment of their competencies (Fig. 2).

**Fig. 2:**
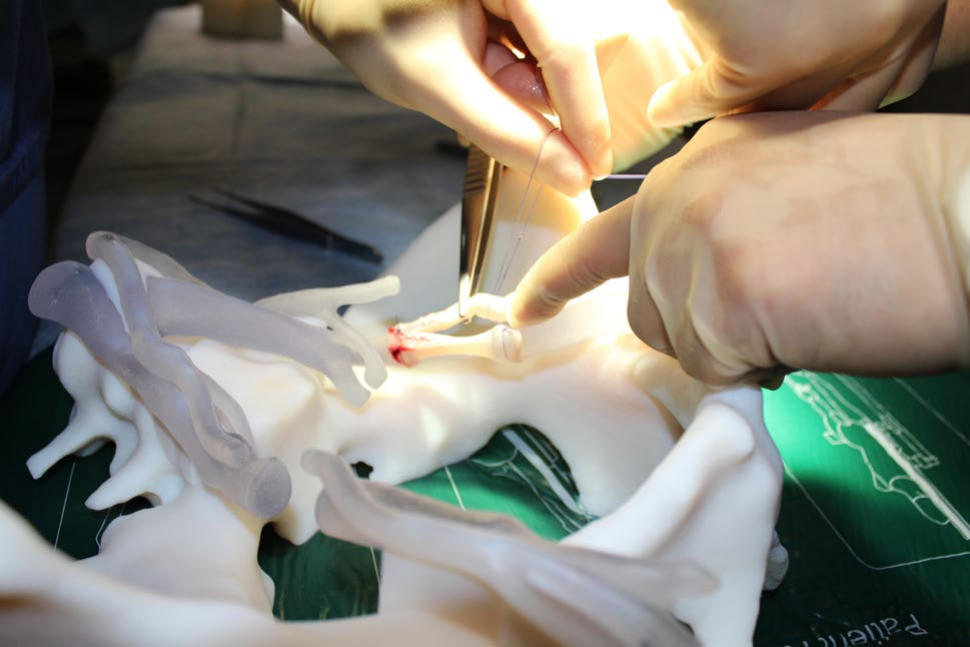
Addition of deceased donor vessels to the cradles

The key concept of this model is the use of human tissue to maximise the fidelity of the simulation exercise. The storage and use of deceased donor vessels for training purposes required institutional review and approval from the legally responsible organization under the United Kingdom Human Tissue Act 2008 legislation, which is NHS Blood and Transplant (NHSBT), was obtained. Eligible vessels for this tissue bank were iliac arteries and veins. They were collected when unused for either liver or pancreas transplants.

The prosthetic component consists of a synthetic 3D printed cradle of an anatomically accurate pelvis printed in life-size. In addition, 3D printing was used to create a kidney and a plinth to support the kidney in the correct operative position within the pelvis.

Recipient anatomy that was reproduced included:

- pelvis
- vertebral column from L4 to coccyx
- abdominal vasculature: aorta and IVC from L4 level,
- iliac vessels: the complete common iliac vessels, the internal iliac vessels terminating 3 cm distal from their origin; external iliac vessels to femoral bifurcation.

Donor anatomy included the body of the kidney and the renal artery and vein with an exposed length of 2 cm.

Deceased donor iliac arteries and veins were obtained from the tissue bank. These were dissected and inter-positioned in the gap of the recipient’s iliac artery and vein and secured with 2.0 Vicryl ligatures. Deceased donor iliac vessels were also mounted in a similar fashion on the 3D printed kidney’s vascular stumps. The da Vinci Si robotic system was successfully docked in situ by the robotic team. Two robotic surgeons performed vascular anastomoses between the ‘hybrid donor’ renal vessels and the ‘hybrid recipient’s’ iliac vessels using 6.0 prolene sutures. Mean anastomotic procedural time was 20 min per vessel. Vascular anastomotic patency was tested by intravascular injection of saline using a hypodermic needle. A good anastomotic patency and leak-resistance was demonstrated. Room and operating field video recording was undertaken for training feedback purposes. This facilitated group discussion, reflection and feedback.

The model offers several advantages. It is anatomically accurate offering geometrical fidelity and realistic spatial constraints. It utilizes deceased donor vessels to replicate reality. Deceased donor vessels also offer the advantage of being pre-screened for infection allowing it to be used in normal clinical operating theatres with the same operating robot used in clinical cases. In addition, the model can be used repeatedly, making it ideal for high-volume training, even in centers without 3D printing facilities. The use of video capture methods allows retrospective analysis of performance and structured debriefing for training, as well as revalidation purposes. Finally, the model can be deployed in situ to aid team-oriented training, as well as enhancing user immersion. Model development costs were acceptable, and the estimated reprint cost is around £1000. This represents a significant financial advantage compared with training on animal models or the cost of consumables in other forms of simulation (Fig. 3).

**Fig. 3:**
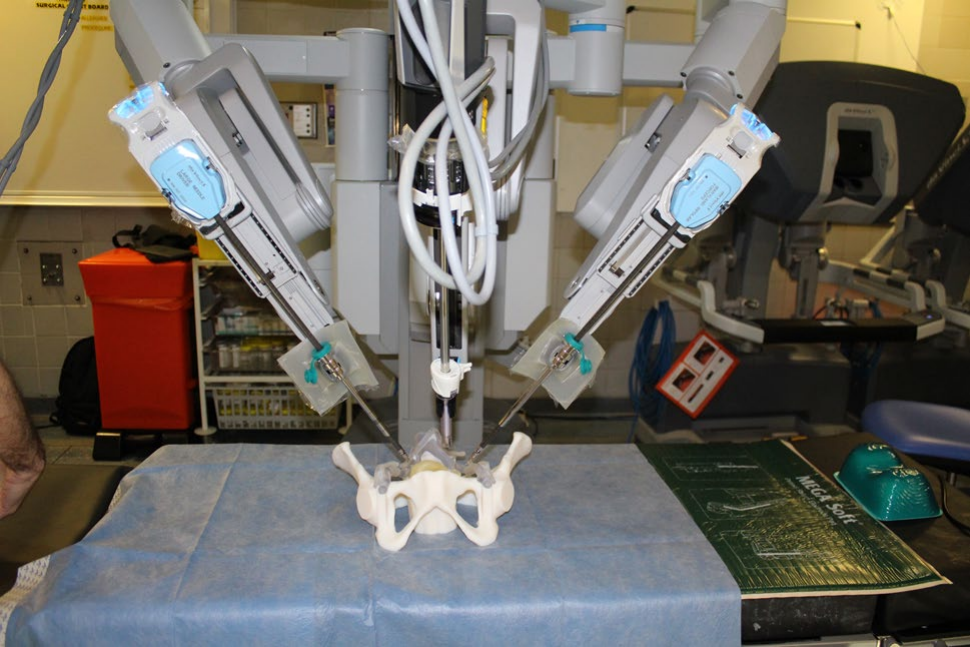
Model in the operating theatre with the operating robot ready to use

In the future, the model offers the opportunity to create bespoke patient-specific training, allowing surgeons to practice on clinically targeted operating fields. Fidelity may be further enhanced using the model in conjunction with an abdominal cavity simulator, which is already available. It has the potential to be incorporated into a future training curriculum for robotic transplant surgery (Fig. 4).

**Fig. 4:**
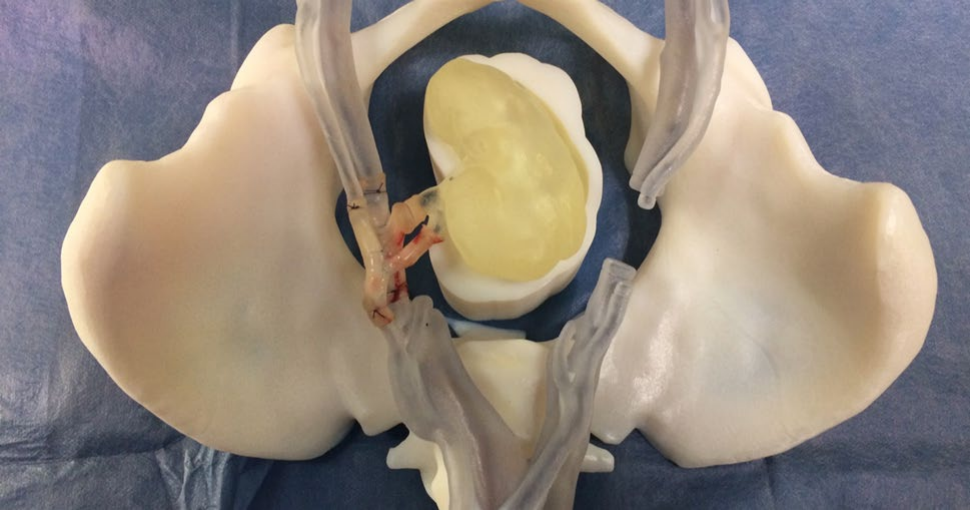
Model with completed anastomoses

Main limitations of this concept include access to 3D printing facilities and deceased donor vessels. Another obstacle is that robotic theatre is usually available during non-office hours.

In summary, the model demonstrates how 3D-printed-enhanced simulation can be used to bridge the gap between standard simulation and clinical practice in a simple and cost-effective manner. This represents a new step in hybrid RAKT simulation, combining novel 3D-printed technology with the time-tested benefits of using deceased donor tissue for high fidelity simulation training. The model is currently under validation by a multidisciplinary team of experts in robotic surgery.
